# Cardiovascular and Neuronal Consequences of Thyroid Hormones Alterations in the Ischemic Stroke

**DOI:** 10.3390/metabo13010022

**Published:** 2022-12-23

**Authors:** Melania Murolo, Olivia Di Vincenzo, Annunziata Gaetana Cicatiello, Luca Scalfi, Monica Dentice

**Affiliations:** 1 Department of Clinical Medicine and Surgery, University of Naples “Federico II”, 80131 Naples, Italy; 2 Department of Public Health, University of Naples “Federico II”, 80131 Naples, Italy; 3 CEINGE-Biotecnologie Avanzate S.c.a.r.l., 80131 Naples, Italy

**Keywords:** thyroid hormones, hemorrhagic and ischemic stroke, myocardial infarction

## Abstract

Ischemic stroke is one of the leading global causes of neurological morbidity and decease. Its etiology depends on multiple events such as cardiac embolism, brain capillaries occlusion and atherosclerosis, which ultimately culminate in blood flow interruption, incurring hypoxia and nutrient deprivation. Thyroid hormones (THs) are pleiotropic modulators of several metabolic pathways, and critically influence different aspects of tissues development. The brain is a key TH target tissue and both hypo- and hyperthyroidism, during embryonic and adult life, are associated with deranged neuronal formation and cognitive functions. Accordingly, increasing pieces of evidence are drawing attention on the consistent relationship between the THs status and the acute cerebral and cardiac diseases. However, the concrete contribution of THs systemic or local alteration to the pathology outcome still needs to be fully addressed. In this review, we aim to summarize the multiple influences that THs exert on the brain and heart patho-physiology, to deepen the reasons for the harmful effects of hypo- and hyperthyroidism on these organs and to provide insights on the intricate relationship between the THs variations and the pathological alterations that take place after the ischemic injury.

## 1. Introduction

Stroke is one of the most frequent neurological disorders leading to chronic disability and is recognized as a major cause of morbidity and mortality worldwide. 

Its onset depends on several risk factors including age, race, and personal habits such as cigarette smoking and alcohol consumption, socioeconomic status and comorbidities like diabetes mellitus, hyperlipidemia and hypertension [1,2,3,4]. Depending on the cause of its insurgence, stroke can be classified in the two following main categories: the hemorrhagic and the ischemic. The former is primarily determined by chronic hypertension and Cerebral Amyloid Angiopathy (CAA), leading to vascular remodeling [5,6]. Moreover, brain aneurysms and ruptured arteriovenous malformation (AVM) can cause cerebral hemorrhages and are associated with increased morbidity and mortality, especially in young individuals [7,8]. Instead, cerebral ischemia is caused by a clot-induced interruption of blood flow triggering hypoxic conditions and nutrient deprivation of the cerebral tissue [9,10,11,12]. The lack of cerebral blood supply induces a massive necrosis and an irreversible neuronal damage that prompts a significant release of glutamate with consequent hyperactivation of the N-methyl-D-Aspartate (NMDA) receptors. The stimulation of the NMDA receptors, in turn, provokes a high Ca^2+^ flow into the cells, thus inducing their death for excitotoxicity [13,14,15]. As cells die, molecular injury signals further enhance neuroinflammation stimulating the cytokines expression and release, generating the additional recruitment of leukocytes from the peripheral blood [16,17]. This feed-forward inflammatory response in the brain also increases the cytokine secretion in circulating blood, thus determining regional and systemic phlogosis that severely impacts the disease outcomes [18,19].

The complex relationship between the thyroid hormones serum concentration and the insurgence, severity and recovery after stroke remains controversial because thyroid hormones exert both neurotoxic and neuroprotective effects [20,21]. Thyroid hormones (THs), the prohormone L-Thyroxine (T4, or 3,3′,5,5′-tetraiodo-L-thyronine) and the active form Triiodothyronine (T3, or 3,3′,5-triiodo-L-thyronine), are pivotal determinants of the energy metabolism and the homeostatic control of basically every body tissue [22,23,24]. While the systemic THs concentration depends on a central control exerted by the Hypothalamus-Pituitary-Thyroid (HPT) axis, their intracellular levels are finely tuned by a complex network of crucial regulators of THs signaling: the plasma membrane transporters, mainly MCT8 and MCT10, which mediate the THs cellular uptake [25]; the different isoforms of THs receptors, TRα and TRβ that, through the binding to THs, regulate the transcription of TH target genes [26], and the deiodinase enzymes [27]. These latter consist of three selenoenzymes (D1, D2 and D3) that catalyze the activation or inactivation of THs and are differentially expressed in various tissues depending on the pathophysiological context [28,29,30]. The type I deiodinase, D1, plays a central role in rT3 clearance and T3 maintenance by catalyzing the deiodination of rT3, its preferred substrate, and T4, to form T2 and T3 [31,32]. Moreover, D1 catalyzes the deiodination of T4 to produce T3, to finely control the systemic T3 physiological levels and sustain the recycling of iodine, maintaining the thyroid reservoir [31,32]. The type II deiodinase, D2, exhibits a higher preference for T4 as the deiodination substrate, thus representing the major T3 supplier for the intracellular milieu to modulate the target genes transcription. Finally, the type III deiodinase, D3, barely expressed in adult tissues, is the main THs inactivator, prompting the conversion of both T4 and T3 in THs’ inactive forms [33].

The deregulation of THs signaling has a strong impact during both development and adult life, representing one of the leading causes of several pathologies, such as neurological cretinism, spastic dysplasia, Alzheimer’s disease, metabolic syndromes, diabetes, obesity, skeletal muscle atrophy and multiple cancer types [29,30,34,35,36,37,38,39,40,41]. The influence of THs on some of these conditions is reflected directly or indirectly on the risk of stroke and its clinical course. As above-mentioned, the major risk factors for the ischemia are represented by metabolic syndrome, diabetes, dyslipidemia and hypertension, all diseases strongly affected by THs. Indeed, these latter are primarily involved in the control of several metabolic pathways related to fatty acid oxidation, fat storage, insulin sensitivity and glucose metabolism. Thus, it is not surprising that several studies indicated that huge variations occur in these contexts in response to a hypo- or hyperthyroid state. In detail, the subclinical and overt hypothyroidism has far been demonstrated promoting obesity, low-density lipoprotein (LDL) and cholesterol accumulation as well as diastolic and systolic blood pressure increase and insulin resistance [42]. All these events predispose to the onset of Type 2 Diabetes mellitus (T2DM) that in turns affects the risk of stroke [43]. For this reason, the therapeutic application of THs mimetics has been reported as a valid option for the treatment of lipids dysregulation and storage, deeply improving hepatic steatosis [42]. On the other hand, a condition of hyperthyroidism equally contributes to the development of pathologies that affect stroke onset such as atrial fibrillation and metabolic alteration in insulin sensitivity. Indeed, albeit more certain for hypothyroidism, multiple reports revealed discrepant results about the contribution of thyrotoxicosis to the occurrence of insulin resistance, primarily inducing hepatic glycogenolysis and downregulating hepatic glycogen synthesis [42]. 

Although, over the years, several literature data have highlighted an effect of the systemic THs alterations on the risk of stroke, neuronal damage and post-injury outcome, the results remain debatable. In the attempt to shed light on the involvement of THs in modulating the different aspects of ischemic diseases and the functional recovery of patients affected by this pathology, this narrative review aims to describe the role exerted by THs in the diverse tissues involved, with particular regard to the effects of hypo- and hyperthyroidism on stroke progression.

## 2. The Deiodinases-Mediated Thyroid Hormones Metabolism in the Brain Physiology and Pathology

Thyroid hormones govern neurological development and physiological brain maturation. Hence, the strict control of THs concentrations is essential in both physiological and pathological conditions, where the regulation of the deiodinases profile expression represents the principal molecular mechanism adopted by cells to orchestrate THs levels.

Since the first weeks of embryonic life, a tight spatial and temporal modulation of deiodinase expression occurs, in order to limit the THs concentration in the maternal blood [44]. In particular, D3 is critically increased during gestation in the placental syncytiotrophoblasts, cytotrophoblasts, in the endothelium of fetal vessels and in the maternal decidua. Such an increase is functional to lower T3 serum concentration of the fetus and favor the implantation [45]. 

In the adult, the regulation of THs levels in the central nervous system (CNS) is ensured by the expression of the THs transporters on the choroid plexus of the blood–brain barrier, which exhibits a greater affinity for T4 with respect to T3 [46]. Once crossed the endothelial cells of brain capillaries, the local availability of THs is regulated by the deiodinases, which are selectively expressed depending on specific cell types. Indeed, D2 and D3 are differentially expressed in astrocytes and neurons: D2 is located in astrocytes to provide an appropriate tissue T3 content, while D3 is expressed in neurons to attenuate the astrocytes-derived T3 and ensure proper nuclear availability of T3 [47]. The balance between the two deiodinases represents a neurological protective mechanism against the harmful effects of hyper- or hypothyroidism, since chronic and severe alterations in THs levels are responsible for cognitive and psychiatric disorders [48].

In accordance with the key role played by the peripheral activation of T4 by the deiodinases, it has been shown that mice lacking D1 and D2 have reduced T3 levels in the brain, as well as reduced expression of several TH-target genes [49,50] and altered motor ability [51] along with enhanced anxiety behavior [52]. Moreover, mice with the reduced D2 activity due to the Ala92-Dio2 polymorphism displayed reduced physical activity, slept more and required additional time to memorize objects, while T3 replacement improved the cognition [53]. Similarly, neuronal-specific inactivation of the thyroid hormone receptor TRα in mice caused enhanced anxiety behavior [54]. All this evidence is in agreement with the clinical observation that hypothyroid patients experience alterations in memory, concentration and psychomotor speed, with increased depressive and anxiety disorders [55,56,57]. Thereby, these data point to a critical role for the local regulation of THs in the brain physiology and suggest that alteration of the THs homeostasis can affect the response of neuronal cells to pathological events such as the stroke. In addition, derangements of THs’ levels are often associated with worsened inflammatory response in different pathological conditions, thus amplifying the harmful effects of THs alteration in the tissue pathology [58,59]. Studies addressing the role played by THs transporters, receptors and deiodinases in the stroke occurrence and progression are still missing and constitute a critical challenge for the future.

## 3. Neuroprotective Actions of Thyroid Hormones in Acute Stroke

The T3-mediated positive effects on brain development result from its dual action on astrocytes and neurons and include the stimulation of neuronal differentiation, axonal maturation, myelination and promotion of synaptic plasticity [60,61]. In vitro experiments showed that, on one hand, T3 enhances astrocytes viability, by inducing glutamate uptake after an excessive glutamate exposure; and on the other, it counteracts the neural death by boosting the mitophagy and promoting the mature neurons-neural stem cells crosstalk after traumatic brain injury [60,62,63]. Confirming these data, several in vivo studies demonstrated the positive effects of T4 and T3 in decreasing neuronal damage, infarct volume after stroke, and inflammatory genes expression, as well as in increasing neuronal survival and neurogenesis [64,65,66,67]. Moreover, THs reduced blood pressure in focal cerebral ischemia, acting on endothelial Nitric Oxide Synthase (eNOS) and stimulating vasodilation [64,65,66,67]. In support of the above-described beneficial role of THs in promoting post-injury brain recovery, Boltzmann et al. observed a drop of serum T3 concentration in patients in the acute phase of the disease, probably due to impaired peripheral conversion of T4 and the marked release of cytokines that inhibit THs metabolism [68]. In line with this evidence, a rat models of middle cerebral artery occlusion showed a reduction of THs serum levels 14 days after injury in concomitance with the onset of neurological deficiency [69]. Finally, the enhanced D2 expression in astrocytes 72 h post-transient cerebral ischemia, along with the reduced TRβ expression in the infarct core, and its increase in the peri-infarct area, suggest a crucial role of THs in regulating astrocytes response to neurological injury [69,70].

## 4. Correlations between Thyroid Hormones Alterations and Stroke

If physiological THs levels are essential to guarantee proper neuronal function in the CNS, low THs levels have harmful effects and represent negative prognostic factors for stroke. Overt hypothyroidism, a clinic syndrome characterized by systemic THs deficiency, has long been associated with neuronal dysfunctions such as depression, dysphoria, attention deficit, cognitive decline, learning and psychomotor performance degeneration, as well as atherosclerosis and increased risk of cerebrovascular accidents [71,72]. Moreover, hypothyroid patients are affected by increased cardiovascular morbidity caused by elevated Low-Density Lipoprotein (LDL) and cholesterol levels, diastolic hypertension and endothelial dysfunction. These alterations result in an enhanced risk of stroke (particularly ischemic stroke), greater initial injury severity, increased rate of in-hospital deaths, as well as poor functional outcome [20,62,73,74,75,76,77,78,79,80,81]. On the contrary, the association between cardiovascular disorders and the hypothyroid state could be attributed to the protective actions of THs on neurons and glial cells against glutamate toxicity, avoiding the downstream effects of the cerebrovascular accident. 

As in the case of hypothyroidism, some epidemiological studies have shown a worsening effect of hyperthyroidism (lower levels of TSH and higher levels of free T4) on stroke prognosis and severity [82]. Probably, the reason behind this association is the influence of THs on the ischemic/reperfusion phenomenon. Indeed, an excessive increase in THs levels corresponds to more intense ischemic-reperfusion injury in human patients [67]. Moreover, the blockage of D2 enzymatic activity by daily intravenous administrations of the inactive rT3 prevented ischemia in animal models [83,84]. These observations indicate that, although crucial for cell physiology, when in excess, THs can exacerbate the sympathetic nervous system effects [85] causing a dangerous hypermetabolic state, characterized by higher production of Reactive Oxygen Species (ROS) and free radicals that lead to cytotoxicity [86]. The exact mechanisms underlying THs-dependent negative repercussions on stroke remain still poorly understood, also in the light of additional effects of hyperthyroidism on other related pathologies such as respiratory distress, cardiovascular diseases and cancer, which can influence the course of the illness in an independent manner [29,87,88].

**Figure 1:**
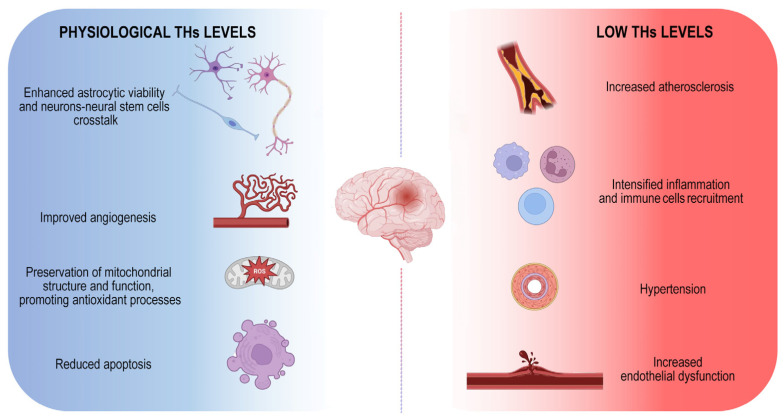
Physiological THs maintenance is essential to counteract ischemic stroke onset. Schematic illustration of positive THs-dependent effects on brain. The image represents the harmful consequences of low THs levels indicating the main mechanisms by which they influence the onset of the disease.

Although both hypo- and hyperthyroidism are associated with an increased risk of stroke, the hypothyroidism is the condition that mostly affects the probability of ischemic events (Figure 1).

## 5. Cerebral Impact of Subclinical Hypothyroidism

**Figure 2:**
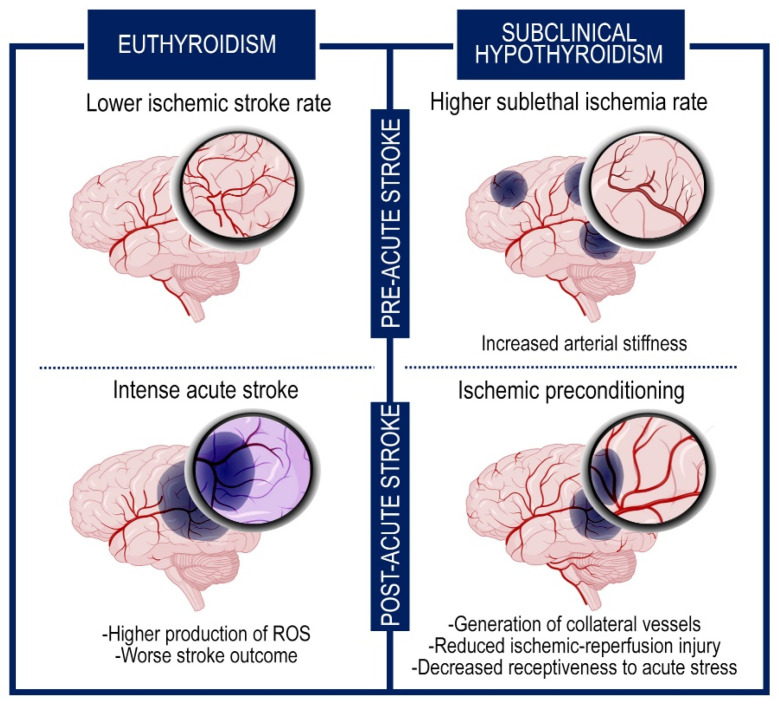
The subclinical hypothyroidism exerts neuroprotective effects through an ischemic preconditioning system. Graphic representation of the brain in subclinical hypothyroidism and euthyroid condition. The figure shows the events that occur before and after the intense ischemic accident. The subclinical hypothyroidism increases the frequency of sublethal ischemia but ameliorates the subsequent response to acute cerebral stroke for the generation of collateral vessels and the hypometabolic rate.

Even if the condition of subclinical hypothyroidism (characterized by high TSH levels but normal T3 and T4 levels) increases the incidence of transient ischemic attacks, recent reports suggest that it has a beneficial impact on preventing post-stroke severe outcome and reducing the mortality rate [89,90]. Multiple suppositions have been put forward to interpret this effect. One explanation might be that the elevated TSH levels can result in an increase of systemic vascular resistance for the strengthened arterial stiffness, that in turn incurs in sublethal ischemia prompting the generation of collateral vessels. These events ameliorate the response to intense cerebral accidents and improve the clinical picture (Figure 2) [76]. 

Besides this endogenous ischemic preconditioning, other processes may cooperate as protective mechanisms against the severe consequences of the ischemic attacks, decreasing the metabolic demand of neurons and the oxidative stress [76,91,92]. Among them, the reduced receptiveness to acute stress, due to the refractoriness to the adrenergic system, and the lower glucose levels, characteristic of hypothyroidism, are associated with a better prognosis in stroke [93].

## 6. Thyroid Hormones and Clinical Outcomes in Post-Stroke Patients

To date, clinical studies on alterations of THs levels and stroke have focused mainly on the correlation between the low T3 syndrome and a worsened clinical outcome. In acute stroke patients, the incidence of low T3 levels varied from 15.1% [94] to 37% [73]. These studies have demonstrated the association between lower T3 levels with greater stroke severity [74,95], infections [96], more complicated in-hospital clinical course [95], greater mortality rates [74,95] and a higher risk of poor functional outcome [73,74,76,77,95,97,98]. Moreover, low T3 syndrome has been reported to affect 32% to 62% of stroke patients in the acute phase [95], and to be associated with a higher prevalence of cognitive impairment post stroke, independently of other risk factors [99]. Furthermore, low T3 syndrome represents a possible risk factor for in-hospital stroke-associated pneumonia [100].

While for ischemic stroke hypothyroidism is associated with a poor clinical outcome [76], in case of haemorrhagic stroke, one study showed that a history of hypothyroidism did not affect clinical severity, mortality or functional outcome [101].

Data on T4 and stroke are conflicting. A meta-analysis by Jiang et al. [77] showed that patients with acute ischemic stroke with a poor outcome had higher T4 levels. Conversely, other findings reported that neither free T4 (FT4) nor TSH levels were associated with a functional outcome [96]. In accordance, hyperthyroidism was associated with an increased risk of ischemic stroke, independent of cardiovascular risk factors [102]. Instead, studies in a large population-based cohort of THs users have demonstrated that both hyper- and hypothyroidism were associated with an increased risk of stroke [103]. In addition, the subclinical form of hyperthyroidism may represent a risk factor for poor outcome three months after ischemic stroke [104]. Finally, a recent study found that high and low T4 levels were associated with lower global brain perfusion compared to middle levels of T4 [105].

Data on the prevalence of low TSH (5.4–32.6%) was reported in a few studies [76,106]. Low TSH was an independent risk factor for mortality [106], poor functional outcome [76,106], and it showed the potential to predict fatigue after acute ischemic stroke [107]. Recently, it was reported that high TSH levels on admission might be associated with mortality [108] and depression in acute ischemic stroke patients [109]. Although some evidence on altered THs concentration are reported in patients in the acute phase of the disease, only one study involved post-acute patients undergoing rehabilitation [68].

## 7. Thyroid Hormones Effects on Acute Heart Disease

THs exert significant cardiovascular effects influencing cardiomyocytes maturation, function and metabolism, via a variety of molecular mechanisms. In the perinatal period, the systemic levels of T3 undergo a dramatic increase that elicits the Myosin Heavy Chain (MHC) switching, the hypertrophy of myofibers, the mitochondrial respiration and the induction of the calcium-ATPase pump of the sarcoplasmic reticulum (SERCA) [110,111]. As a result, abnormalities in fetus THs levels entail a series of anomalies including stunting, heart output reduction, atrial fibrillation and abortion [112]. During adult life, THs control myocardial contractility and structure, and impact the electrophysiological activity and cardiac metabolism. Indeed, acting through genomic and non-genomic mechanisms, they regulate the vascular smooth muscle cells tone and modify the membrane ion channels profile, boosting the systolic artery pressure and increasing the cardiac rhythm [24,113]. Furthermore, THs stimulate the metabolic rate promoting mitochondriogenesis and aerobic metabolism, lipogenesis, gluconeogenesis and the amino acids uptake [113,114,115]. Considering the plethora of biological TH-regulated functions, alterations in THs status have the natural consequence of raising different and severe morbidities, primarily myocardial stroke and heart failure [116]. Accordingly, a concomitant alteration in the ratio between TRα1 and 2 isoforms was observed in failing human hearts, with a higher expression of the transcript of the dominant negative variant TRα2 to the detriment of TRα1, that, instead, mediates the positive effects of THs on transcription regulation [117]. Furthermore, during myocardial infarct, it has been described a significant stimulation of D3 activity, probably caused by the chronic inflammation promoted by the Tumor Necrosis Factor- α (TNF-α) and the Interleukin-6 (IL-6) release, thus complicating the hypothyroid condition. Indeed, in individuals with acute myocardial infarction, the rise in IL-6 is tightly related to a drop in T3, due to the inhibitory activity that IL-6 exerts on D2 [118]. This trend determines an MHC shift in favor of the beta isoform, resulting in a myosin V3 isoenzymes (constituted by homodimers of two MHCβ) cardiac predominance and in the downregulation of the SERCA2 pump [118]. The transition in the myosin content is responsible for a lower myosin ATPase activity and consequently for a reduced contraction velocity, which in turn affects the cardiac function under stress conditions and contributes to the onset of the heart disease [119]. The variation in the MHC isoforms distribution can be reverted by T3 and T4 supplementation, as well documented in in vivo experiments and human studies, where high TSH and low T3 and T4 levels were found associated with maladaptive cardiac decompensations [117,119]. The positive effects exerted by THs are also extended to the metabolic changes that occur during the ischemic myocardial insult. Throughout the ischemic episode, there is a strong arrest of the blood nutrient supply that triggers hypoxic conditions and rapidly induces metabolic changes, such as the repression of the oxidative metabolism and the boosting of the anaerobic glycolysis [120]. Following the injury, the recovery of the blood flow prompts a further microvascular damage caused by the tissue reperfusion. This event determines the abrupt rescue of the oxygen content, leading to an aberrant response consisting in the generation of ROS, endoplasmic reticulum stress, mitochondrial dysfunction and immunocytes recruitment [121]. One of the most critical end-effectors of ischemia/reperfusion-induced damage is the opening of mitochondrial pore that induces cell lysis and apoptosis [121]. In this context, it has been widely demonstrated the pro-survival, antioxidant and proangiogenic effects of THs [30,118,122,123]. Indeed, they decrease the activation of the pro-apoptotic p38-MAPK, the tumor suppressor p53, the pro-apoptotic Bax, cleaved caspase 3 and 9, and upregulate the expression of the anti-apoptotic factor Bcl-2, the proangiogenic HIF-1α and the antioxidants Nrf-2 and HO-1, reducing the cardiac scar area and promoting the new vessels formation and the cell viability [118,124]. These effects culminate in a cardiac preconditioning system that protects the infarcted heart from the major adverse cardiac events, attenuating the cardiogenic shock and death rate [118]. Besides the favorable actions that physiological THs levels exert in this context, overt and subclinical hyperthyroidism have been widely described as predisposing conditions of atrial fibrillation, a major stroke risk factor [125]. Conversely, the data about the influence of hypothyroidism on this event led to uncertain and conflicting results [125,126,127,128]. In fact, the effects of the low THs levels on the risk of myocardial infarction are certainly more recognized on other aspects, such as atherosclerosis and diastolic hypertension [129]. Although the consequences of the above-mentioned thyroid dysfunctions have been partially addressed for what concerns the risks, the debate on the relationship between the THs systemic levels and the heart stroke outcome is still opened. While there is a robust association between decreased TSH levels and hyperthyroidism with higher odds of atrial fibrillation [130], any statistically significant and consistent associations were observed between TSH levels, hyperthyroidism or hypothyroidism, or FT4 levels with the other cardiovascular outcome. Only a few evidence suggested a possible association between decreased TSH levels and higher odds of cardioembolic stroke, hyperthyroidism and lower probability of thoracic aortic aneurysm events [131].

Multiple studies have highlighted the correlation between the circulating T3 levels and the recovery ability of the ventricular function, indicating T3 as a significant predictor of myocardial ischemia outcome [132,133,134,135]. Accordingly, patients with elevated systemic rT3 or low T3 have been reported more prone to short and long-term mortality, unsuccessful cardiac rehabilitation and rising blood levels of indicators for acute cardiac distress, such as troponin T and N-terminal pro-brain natriuretic peptide [118,122,136]. Moreover, an inverse correlation exists between the reduction of serum T3 levels and the severity of the cardiac disease [137].

In light of the control exerted by T3 in myocardial contractility and metabolism, and of the numerous evidence that demonstrated an important link between THs concentration and the heart stroke gravity, incidence, and recovery post-injury, THs can be considered important predictive biomarkers and major players in determining the acute coronary disease outcome.

## 8. Discussion

Central and local thyroid hormones dysfunctions are frequent conditions reported in case of ischemic events. THs are involved in several physiological processes and, in particular, are important players in driving the development and the maturation of multiple tissues through genomic and non-genomic actions [23,24,67,92,115,123]. It has long been known that THs have a variety of effects on the cardiovascular system and brain development. Maintaining optimal THs levels is essential to ensure whole body homeostasis and to guarantee brain and cardiovascular function throughout life. The mechanisms by which THs mediate their effects on the cardiovascular system and the brain overlap with those recognized to improve the recovery of physiological functions after stroke. Thereby, alterations of such TH-dependent mechanisms affect the outcome of both cerebral and cardiac diseases.

Consequently, it is not surprising that disruptions in THs availability and function may result in significant variations in injury outcome. However, the impact of peripheral and systemic hypo- or hyperthyroidism can strongly differ depending on the type of pathology and, even within the same disease, distinct effects can occur in the context of specific tissues according to the function they exert. 

Although several studies have indicated a relevant association between THs and outcome after stroke, the potential contribution of thyroid dysregulation to the pathogenesis of cardiac and brain failure remains poorly understood.

Significant knowledge gaps exist regarding the precise TH-mediated molecular and biochemical mechanisms that control brain and cardiac physiopathology and the optimal strategies for the management of thyroid dysfunction in patients with and without pre-existing cardiovascular disease. Specifically, although overt hypothyroidism and hyperthyroidism need to be treated in patients with established heart failure, it remains unclear whether subclinical thyroid dysfunction requires similar attention. The lack of epidemiological studies of cohort patients with subclinical thyroid dysfunction limits our understanding of the mechanisms by which subclinical hypothyroidism and the low T3 syndrome increase the risk of mortality in patients with established heart failure. Even in euthyroid patients, the impact of altered myocardial TH actions on heart failure remains unclear. Indeed, the specific TH-mediated myocardial effects that significantly affect the development of human heart failure are still poorly understood and their discovery may shed light on new targets for therapy with T4, T3 or thyroid hormone analogs. 

In the humans, the preservation of THs homeostasis controlled by the HPT system is combined with adaptive mechanisms in non-thyroidal organs that produce their own free T3 levels. Such intra-tissue TH production is also more advantageous than central regulation, because it provides peripheral organs with a certain level of regulatory autonomy to produce more or less THs to meet their different energetic, functional and metabolic needs. For this reason, the accurate quantification of TH action at tissue-level also remains a challenge. Although serum TH levels can be easily measured, there are currently no methods for their routine quantification within tissues, which may be more tightly correlated to myocardial function. Indeed, it is also not clear how TH transporters and receptors are regulated in animal models and in patients with heart failure, and there are no data to fully explain the THs effects on cardiomyocyte contractility and electrophysiology.

On the basis of this multifaceted lack of awareness, areas of particular interest for further research include exploring the fundamental biology linking thyroid dysfunction to the development of cardiovascular disease and identifying novel biomarkers of THs action in the cardiac tissue.

Moreover, large epidemiological studies are needed to define the subgroups of patients with thyroid dysfunction who are susceptible to cardiovascular disease and who can be treated for specific prevention. In addition, clinical trials should focus on evaluating the ability of TH or thyromimetics to improve cardiovascular performance and outcomes. Clinical and preclinical studies will clarify the role of TH in modulating endothelial function and define the cellular signaling processes by which TH regulates electric conduction, contractility and peripheral vascular function. Future research will elucidate the mechanisms underlying atrial and ventricular arrhythmias or myocyte remodeling and neuronal dysfunctions orchestrated by TH that can be exploited to improve the functional recovery in stroke patients.

## 9. Conclusions

In conclusion, according to the available evidence and considering the vital role that THs wield in the pathophysiology of the organs affected by acute ischemia, THs regulation represents a viable therapeutic strategy for stroke management and functional recovery. 

Although with several discrepancies, the wide array of the mentioned studies seems to demonstrate that both conditions of hyper- and hypothyroidism, as well as the low T3 syndrome, are all associated with a poor outcome and act as adverse prognostic factors that negatively affect the regeneration process after acute stroke.

However, albeit the positive effects of THs on tissue recovery are well described, still a few studies have addressed how the THs status impacts the rehabilitation of stroke patients. These studies are of crucial need and might add critical insight, considering the known positive effects exerted by THs on cardiac muscle physiology, brain regeneration and angiogenesis.
